# From vesicle to cytosol

**DOI:** 10.7554/eLife.38847

**Published:** 2018-06-27

**Authors:** Michael J Rogers, Marcia A Munoz

**Affiliations:** 1 Bone Biology DivisionGarvan Institute of Medical ResearchDarlinghurstAustralia

**Keywords:** mechanism of action, membrane transporter, lysosomes, genome-wide screening, bone-targeting drugs, Human, Mouse

## Abstract

Drugs called bisphosphonates are used to treat a range of bone diseases, but how do they reach the enzymes that are their target?

**Related research article** Yu Z, Surface LE, Park CY, Horlbeck MA, Wyant GA, Abu-Remaileh M, Peterson TR, Sabatini DM, Weissman JS, O'Shea EK. 2018. Identification of a transporter complex responsible for the cytosolic entry of nitrogen-containing-bisphosphonates. *eLife* **7**:e36620. doi: 10.7554/eLife.36620

Despite its appearance, bone is a highly metabolic and dynamic tissue that is composed of a vast network of cells called osteocytes that are embedded in a matrix made mostly of collagen and various salts of calcium and phosphate. These osteocytes sense regions of damaged or weakened bone, and 'instruct' bone-destroying cells (called osteoclasts) and bone-forming cells (osteoblasts) to, respectively, remove old bone and deposit new bone (Figure 1). Hence, like a team of road-repairers, the osteocytes, osteoclasts and osteoblasts work together to repair bone and maintain our skeleton in good health (Crockett et al., 2011).

**Figure 1.**
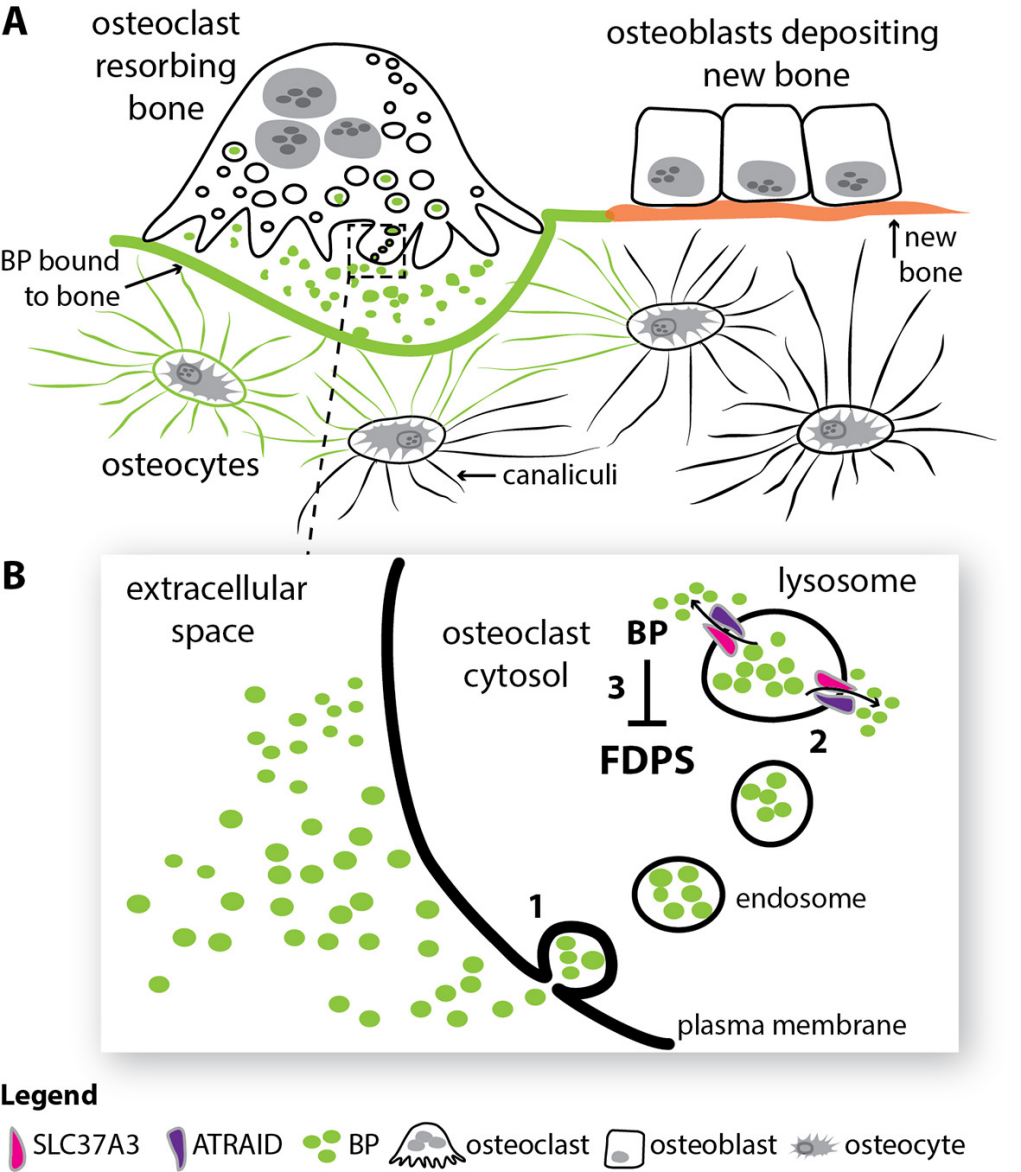
How bisphosphonates act in bone. (**A**) Old and damaged bone is constantly being broken down by cells called osteoclasts in a process called resorption (top left), while new bone is deposited by cells called osteoblasts (top right). Cells called osteocytes (bottom) influence both of these processes through a spidery system of tiny canals called canaliculi. After entering the circulation, the drug bisphosphonate (green) binds very effectively to calcium ions on the bone mineral surface. During resorption, the bisphosphonate on the bone surface is released into the acidic extracellular space beneath the osteoclast. (**B**) The bisphosphonate in the extracellular space is engulfed into osteoclasts via a process called endocytosis (1). The resulting endosomes mature to form structures called lysosomes, and two proteins, SLC37A3 and ATRAID, then interact in the membrane of the lysosome to allow the bisphosphonate to enter the cytosol (2). Once in the cytosol, the nitrogen-containing bisphosphonates inhibit an enzyme called FDPS and prevent the osteoclast from breaking down bone (3). BP: bisphosphonate; FDPS: farnesyl diphosphate synthase; SLC37A3: solute carrier family 37 member A3.

In young adult life there is usually a balance between the amount of old bone broken down and the amount of new bone formed by this repair process, so there is no net gain or loss of bone mass. However, in diseases that affect the skeleton, such as post-menopausal osteoporosis or cancers growing in bone, this delicate balance can be disturbed by osteoclasts being over-active, which leads to excessive bone destruction and fractures. Drugs called bisphosphonates – which inhibit osteoclasts – have been used for more than three decades to treat such diseases and protect the skeleton from potentially catastrophic bone loss, although researchers still do not fully understand how they work. Now, in eLife, Erin O'Shea of Harvard University and the Howard Hughes Medical Institute (HHMI) and colleagues – including Zhou Yu as first author – report the answer to one of the remaining questions about these drugs (Yu et al., 2018).

Bisphosphonates are synthetic molecules that closely resemble the chemical structure of pyrophosphate, which is a natural by-product of numerous metabolic reactions. Importantly, bisphosphonates have two negatively-charged phosphonate groups that enable them to bind calcium ions very effectively, and hence to localize rapidly to any exposed calcium on the bone surface (Rogers et al., 2011). The mechanisms used by bisphosphonates to inhibit osteoclasts remained a mystery for several decades after they were first used in the clinic, but this did not stop the development of improved versions of the drugs (Russell et al., 2008). Eventually it was discovered that nitrogen-containing bisphosphonates (N-BPs), which are now widely used to treat osteoporosis and other bone diseases, work by inhibiting an enzyme called FDPS inside the osteoclasts (Luckman et al., 1998; Bergstrom et al., 2000; Dunford et al., 2001). The N-BP molecules displace the lipid substrates that the FDPS enzyme usually acts on, locking the enzyme in an inactive state (Kavanagh et al., 2006; Rondeau et al., 2006). Without FDPS activity, osteoclasts are no longer able to degrade bone (Rogers et al., 2011).

However, one question remained: how do the N-BPs and other bisphosphonates actually reach the FDPS enzyme, which is in the cytosol of the osteoclasts? There was little or no evidence that a receptor on the plasma membrane was involved (Thompson et al., 2006). Studies with fluorescently-tagged bisphosphonates showed that they first entered the osteoclasts via endocytosis – a process that involves the cell membrane folding inwards and then pinching off to create a vesicle inside the cell (Coxon et al., 2008). But how do the drugs leave these vesicles – which are enclosed by a membrane – to enter the cytosol? Bisphosphonate molecules have a large negative charge, which rules out passive diffusion across the vesicle membrane, which in turn suggests the possibility of a hitherto unidentified transport mechanism (Thompson et al., 2006).

Yu et al. – who are based at Harvard, HHMI, UCSF, MIT, the Broad, Koch and Whitehead Institutes, and Washington University – report that they have identified a protein called SLC37A3 that is required for the release of N-BP molecules from vesicles into the cytosol. Using a CRISPR-based approach to screen for genes that, when missing, confer resistance to bisphosphonates, they identified *SLC37A3* as the gene with the strongest effect. Although the exact function of the SLC37A3 protein remains to be clarified, related members of this protein family are involved in the transport of charged molecules across membranes (Cappello et al., 2018).

Yu et al. found that SLC37A3 interacts and co-localizes with a protein called ATRAID at the vesicle membrane (Figure 1). Importantly, vesicles isolated from cells that did not express *SLC37A3* or *ATRAID* appeared unable to release N-BP molecules, and these cells were much less sensitive to the pharmacological effect of N-BPs.

This new transport mechanism identified by Yu et al. raises interesting questions about how the SLC37A3/ATRAID complex specifically recognizes N-BP molecules, and how it transports them across the membrane of the vesicle. It will also be worthwhile to determine whether differences in the expression of *SLC37A3* or *ATRAID* account for the different sensitivity of osteoclasts and other cell types to N-BP molecules, or whether variants in these genes affect the clinical responsiveness of patients to these drugs. Nevertheless, these elegant studies explain how negatively-charged N-BP molecules can gain access to the cell cytosol after endocytosis and, as a result, go on to benefit huge numbers of patients with potentially devastating bone diseases.
